# Perioperative Immunonutrition for Elective Surgery: Review of Mechanisms, Summary of Evidence, and Future Directions

**DOI:** 10.3390/nu18101603

**Published:** 2026-05-18

**Authors:** Laura Perez Arteaga, D. Dante Yeh

**Affiliations:** 1 Department of Surgery, University of Colorado School of Medicine, Aurora, CO 80045, USA; laurac.perez@outlook.com; 2 Department of Surgery, Denver Health Medical Center, Denver, CO 80204, USA

**Keywords:** immunonutrition, perioperative nutrition, surgical outcomes, pharmaconutrition, glutamine, arginine, omega-3 fatty acids, ESPEN guidelines, elective surgery

## Abstract

Surgical trauma triggers a complex metabolic and inflammatory response that can profoundly affect patient recovery and clinical outcomes. The magnitude of this stress response correlates with surgical complexity, patient comorbidities, and baseline nutritional status. Immunonutrition refers to the strategic use of specific nutrients, including arginine, glutamine, omega-3 polyunsaturated fatty acids, and nucleotides, to modulate immune and inflammatory responses beyond their basic nutritional value. These pharmaconutrients possess distinct immunomodulatory properties that may attenuate surgical stress responses, enhance immune function, and improve clinical outcomes. While numerous randomized controlled trials and meta-analyses have been conducted over the past several decades, significant methodological heterogeneity, variable product compositions, inconsistent administration protocols, and divergent patient populations complicate the interpretation of findings. Recent well-designed investigator-initiated trials without industry funding have failed to demonstrate benefits, raising questions about the validity of earlier positive findings and challenging current guideline recommendations. The evidence base reveals promising signals of benefit in selected populations combined with substantial limitations. Available evidence suggests that immunonutrition may be most beneficial in severely malnourished patients undergoing major gastrointestinal surgery, particularly when administered perioperatively or postoperatively, though the certainty of this evidence remains moderate at best, given the methodological limitations in the existing literature. From an economic perspective, immunonutrition may represent a dominant intervention in appropriate patient populations, though cost-effectiveness estimates derive primarily from older studies with methodological limitations. However, focus on specialized immunonutrition should not distract from fundamental perioperative nutritional care, including systematic risk screening, optimization when feasible, early postoperative feeding, and achievement of adequate protein and calorie targets. Only through methodologically rigorous research addressing fundamental questions can the promise of perioperative immunonutrition be fully realized.

## 1. Introduction

Surgical trauma triggers a complex metabolic and inflammatory response that can profoundly affect patient recovery and clinical outcomes [1]. The magnitude of this stress response correlates with surgical complexity, patient comorbidities, and baseline nutritional status. During the perioperative period, patients experience increased energy expenditure, accelerated protein catabolism, immune dysfunction, and heightened susceptibility to infectious complications. These physiologic derangements are particularly pronounced in malnourished patients, who face substantially elevated risks of postoperative morbidity and mortality [2].

Traditional perioperative nutritional support has focused primarily on providing adequate calories and protein to prevent further macronutrient deterioration and to support wound healing [2]. However, over the past few decades, attention has shifted toward “immunonutrition”, the strategic use of specific nutrients at supraphysiologic pharmacologic doses that modulate immune and inflammatory responses beyond their basic nutritional value [3,4,5]. These pharmaconutrients, including arginine, glutamine, omega-3 polyunsaturated fatty acids, and nucleotides, possess distinct immunomodulatory properties that may attenuate surgical stress responses, enhance immune function, and improve clinical outcomes [3,4].

The distinction between immunonutrition and standard nutritional support lies in the intended mechanism of action rather than the provision of calories and protein. Standard perioperative nutrition aims to correct macronutrient deficits, prevent catabolism, and support wound healing through adequate energy and nitrogen delivery. Immunonutrition, by contrast, employs specific substrates at doses that exceed ordinary dietary requirements, often referred to as pharmacologic or supraphysiologic doses, with the explicit goal of modulating immune and inflammatory pathways. The concept has its roots in observations from the 1970s and 1980s that individual nutrients such as arginine and glutamine could influence lymphocyte proliferation, macrophage function, and gut barrier integrity independently of their caloric contribution. These findings prompted the development of multicomponent enteral formulas combining arginine, glutamine, omega-3 fatty acids, and nucleotides, which were first tested in trauma and critically ill populations in the early 1990s. Positive results from those early trials generated broad enthusiasm and drove rapid adoption in surgical practice, ultimately leading to guideline endorsement—a trajectory that predated the rigorous methodological scrutiny that characterizes the current evidence landscape.

**Figure 1:**
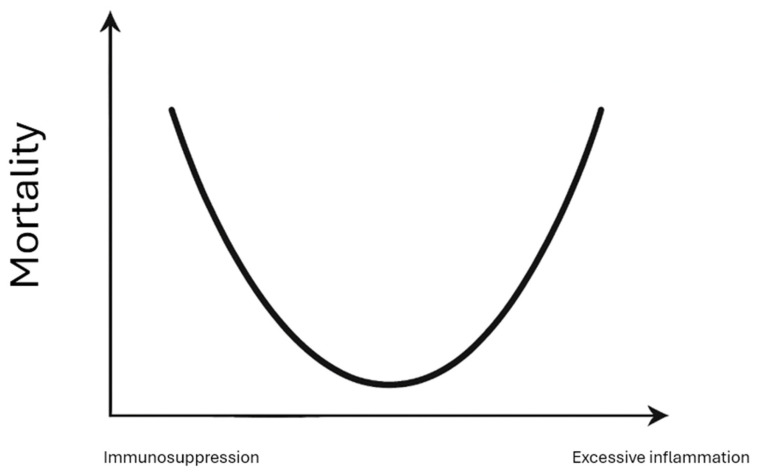
The relative relationship between inflammation and mortality. This schematic illustrates the concept that both insufficient immune response (immune suppression, predisposing to infection) and excessive immune activation (systemic inflammatory response syndrome, predisposing to organ failure) are associated with increased morbidity and mortality. Immunonutrition is hypothesized to modulate this response toward an immunological equilibrium. The x-axis represents the degree of immune/inflammatory activation, and the y-axis represents associated mortality risk, with a U-shaped curve demonstrating that extremes on either end confer excess risk.

The concept of immunonutrition emerged from basic science investigations demonstrating that specific nutrients influence immune cell function, inflammatory mediator production, and tissue repair mechanisms. It is believed that administering immunonutrition may mitigate an excessive hyperinflammatory response to surgical stress or injury, possibly avoiding the common systemic inflammatory response syndrome (SIRS) response and its attendant complications [6]. Conversely, it is also plausible that administering immunonutrients may augment a weakened immune system to improve wound healing and reduce the incidence of infections (Figure 1). Early clinical trials in critically ill and surgical populations generated enthusiasm by showing reductions in surrogate immune function markers, infectious complications, organ failure, and hospital length of stay [7,8]. This promising evidence led to the incorporation of immunonutrition recommendations into major clinical practice guidelines, including those from the European Society for Clinical Nutrition and Metabolism (ESPEN) and the American Society for Parenteral and Enteral Nutrition (ASPEN) [9,10].

Despite widespread guideline endorsement, the clinical application of perioperative immunonutrition remains controversial. The accumulating evidence presents a complex and sometimes contradictory picture. While numerous randomized controlled trials (RCT) and meta-analyses have been published, significant methodological heterogeneity, variable product compositions, inconsistent administration protocols, and divergent patient populations complicate interpretation [11,12] (Table 1). Selective reporting, publication bias, and industry bias have been detected and undermine the confidence in the existing literature [13]; one study reported that an industry-funded RCT is seven times more likely to report a positive result [14]. More recently, well-designed investigator-initiated trials without industry funding have failed to demonstrate benefits, raising questions about the validity of earlier positive findings and challenging current guideline recommendations [15].

This review critically examines the current state of evidence for perioperative immunonutrition in elective surgical patients. Although we will briefly mention immunonutrition in critical illness and trauma, the focus of this article will be elective surgery. Where data from critically ill populations are referenced, this is done specifically to contextualize safety boundaries and the evolution of current clinical practice and not to draw direct equivalence to elective surgical patients. We begin by reviewing the mechanisms of action of individual immunomodulating nutrients to establish the biological plausibility for clinical benefits. We then synthesize findings from landmark randomized controlled trials and meta-analyses to assess the overall efficacy of immunonutrition. Importantly, we analyze methodological flaws and knowledge gaps that limit confidence in existing evidence. Finally, we propose specific recommendations for future research to address these limitations and provide practical guidance for clinical practice. Our goal is to provide clinicians and researchers with a balanced, evidence-based perspective on this evolving field to inform both clinical decision-making and future investigational priorities.

This is a narrative review. A literature search was conducted using PubMed/MEDLINE, Embase, and the Cochrane Library from inception through December 2025 using the following terms: “immunonutrition”, “immune-enhancing diet”, “pharmaconutrition”, “perioperative nutrition”, “arginine”, “glutamine”, “omega-3 fatty acids”, “elective surgery”, and “randomized controlled trial” in various combinations. Priority was given to randomized controlled trials, systematic reviews, meta-analyses, and current clinical practice guidelines. Additional references were identified from the bibliographies of the retrieved articles. No language restriction was applied. Article selection was based on relevance to the clinical questions addressed in this review, with an emphasis on studies in elective surgical populations.

## 2. Mechanisms of Action of Individual Immunomodulating Pharmaconutrients

### 2.1. Glutamine

**Figure 2:**
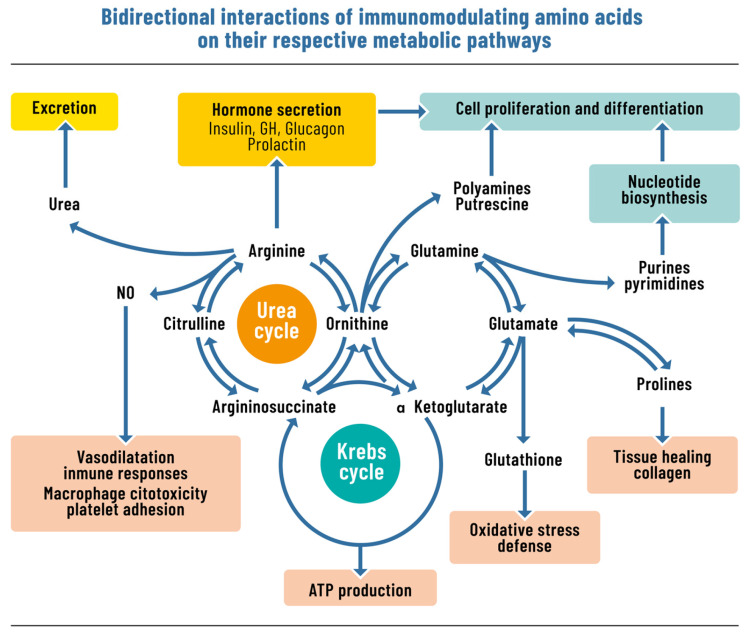
Immunomodulating roles of glutamine and arginine. Glutamine (left pathway): serves as the primary fuel for enterocytes, lymphocytes, and colonocytes; provides nitrogen for nucleotide synthesis; supports glutathione synthesis and antioxidant defense; modulates NF-κB to reduce pro-inflammatory cytokine transcription; and preserves intestinal barrier tight junction integrity, limiting bacterial translocation. Arginine (right pathway): the sole substrate for nitric oxide synthase (NOS), generating nitric oxide (NO) with antimicrobial and immune-signaling properties; metabolized to ornithine for proline/collagen synthesis and polyamine production; restores T-cell receptor expression and IL-2 production; promotes macrophage polarization toward the wound-healing M2 phenotype; and stimulates release of anabolic hormones (growth hormone, IGF-1, insulin). NF-κB: nuclear factor kappa-light-chain-enhancer of activated B cells; NO: nitric oxide; NOS: nitric oxide synthase; IGF-1: insulin-like growth factor-1; IL-2: interleukin-2.

Glutamine is the most abundant free amino acid in the human body and is involved in myriad physiological regulatory pathways, including acid–base balance, nitrogen transport, and cell proliferation, especially in the immune system and gastrointestinal tract [16,17] (Figure 2). While the body can normally synthesize adequate amounts under physiological conditions, endogenous production becomes insufficient to meet the dramatically increased demands that occur during critical illness, major surgery, and trauma. During these periods of metabolic stress, glutamine consumption exceeds the body’s synthetic capacity, suggesting the need for exogenous supplementation and leading some to label glutamine as “conditionally essential” during surgical stress [18,19,20], though some have questioned this label [20].

Glutamine serves as a primary fuel for enterocytes, colonocytes, and lymphocytes, with consumption rates equal to or exceeding glucose utilization. Beyond its role as cellular fuel, glutamine provides nitrogen for nucleotide synthesis, which is essential for immune cell proliferation. During severe illness, skeletal muscle acts as the primary glutamine supplier while the liver consumes it for the urea cycle, converting glutamine to glutamate and NH_3_ via glutaminase for ammonia detoxification. Glutamine provides glutamate for glutathione synthesis, supporting antioxidant defenses and protecting against oxidative injury.

Additionally, glutamine modulates NF-κB by reducing IκB ubiquitinylation, thereby limiting pro-inflammatory gene transcription, and reduces ubiquitin-dependent proteolysis by decreasing ubiquitin gene expression. Glutamine also augments neutrophil function, reduces TNF-α and IL-6 production, enhances IL-10 secretion, improves total lymphocyte counts and percentage of activated CD4 + DR+ T helper lymphocytes, and facilitates earlier normalization of IL-2 levels [21,22]. Animal studies have demonstrated that supplemental glutamine enhances bacterial clearance in an animal model of peritonitis [23].

The preservation of intestinal barrier function represents one of glutamine’s most clinically relevant mechanisms. This amino acid supports tight junction integrity and enterocyte proliferation, preserving intestinal barrier function and reducing bacterial translocation. Glutamine also regulates intracellular heat shock protein expression through HSF-1 activation, protecting cells from stress-induced injury [3]. Additionally, glutamine has been shown to restore intestinal blood flow after resuscitation from hemorrhagic shock, aiding in the preservation of mucosal integrity.

In summary, glutamine is involved in immune cell proliferation and antioxidant defenses while also mitigating pro-inflammatory gene expression and supporting intestinal barrier integrity. Although glutamine was liberally prescribed in previous years, two large, well-designed, and well-conducted trials (REDOX [24] and MetaPLUS [25]) in critically ill patients with multiorgan failure demonstrated no benefit, and, in some patients, glutamine administration resulted in increased mortality. In the post-REDOX/MetaPLUS era, glutamine is seldom given as a separate high-dose parenteral supplement and is usually given in an enteral form as a multicomponent of a bundle of immunonutrients [26]. The most recent European guidelines, developed by a multidisciplinary panel of internationally recognized experts using GRADE methodology, strongly recommend against parenteral glutamine supplementation in critically ill patients with organ failure and state that “additional enteral glutamine shall generally not be given” [27]. As an isolated single pharmaconutrient, glutamine has not been shown to be beneficial in elective cancer surgery patients [28].

### 2.2. Arginine

Arginine, primarily synthesized in humans in the kidney from citrulline or glutamine and classified as a semi-essential amino acid [29], has also been labeled as “conditionally essential” during surgical, traumatic, or septic stress when endogenous synthesis becomes inadequate to meet increased physiological demands [30,31,32,33,34,35]. As the sole substrate for nitric oxide synthase, arginine generates nitric oxide (NO) [32,36], which possesses antimicrobial properties and functions in immune signaling. During arginine depletion, cells generate superoxide instead of NO, causing oxidative injury rather than beneficial signaling (Figure 2).

The inflammatory response to surgery triggers increased arginase activity, which depletes arginine and impairs T-cell function. Arginine supplementation restores T-cell receptor expression, IL-2 production, and Th1 differentiation [33]. Furthermore, arginine facilitates macrophage polarization, promoting the transition from the pro-inflammatory M1 phenotype toward the M2 phenotype that supports wound healing. Exogenous arginine administration has also been demonstrated to enhance macrophage and neutrophil function [34,35,37]. Beyond immune modulation, arginine metabolizes to ornithine, which converts to proline for collagen synthesis and polyamines for cell proliferation [38]. Arginine also stimulates the release of growth hormone, IGF-1, and insulin, anabolic hormones that promote tissue growth [37,39].

Arginine exerts anti-inflammatory effects through NF-κB inhibition, decreasing the production of IL-6, IL-18, and TNF-α. It also inhibits IL-2 and interferon-gamma secretion and blocks adhesion molecule expression. While theoretical concerns exist about NO-induced hypotension in septic patients [35,39,40,41], these have not materialized in clinical trials of elective surgical patients receiving arginine-containing immunonutrition at recommended doses. The 2016 ASPEN guidelines reflect this distinction, recommending against arginine-containing immune-modulating formulas in patients with severe sepsis while suggesting their routine use in surgical ICU patients requiring enteral nutrition [42]. The differential responses between surgical trauma patients and septic patients likely reflect fundamental differences in arginine metabolism and the inflammatory milieu between these populations.

### 2.3. Omega-3 Polyunsaturated Fatty Acids (Fish Oil)

Eicosapentaenoic acid (EPA) and docosahexaenoic acid (DHA) exert anti-inflammatory effects through multiple interconnected mechanisms. When consumed in adequate amounts, EPA and DHA incorporate into cellular membrane phospholipids, displacing arachidonic acid and shifting eicosanoid production from highly pro-inflammatory mediators (PGE_2_, TXA_2_, LTB_4_) to less inflammatory mediators (PGE_3_, TXA_3_, LTB_5_) [43]. This biochemical shift fundamentally reduces the inflammatory potential of the lipid mediator pool.

Beyond producing less inflammatory eicosanoids, EPA and DHA serve as precursors for specialized pro-resolving mediators, including resolvins, protectins, and maresins. These mediators actively promote inflammation resolution by reducing neutrophil infiltration and enhancing macrophage-mediated debris clearance. At the transcriptional level, omega-3 fatty acids activate PPARs (peroxisome proliferator-activated receptors) and inhibit NF-κB, reducing TNF-α, IL-1β, and IL-6 production. This transcriptional reprogramming decreases the expression of multiple cytokines and adhesion molecules [44]. Omega-3 fatty acids also modulate intracellular signaling by reducing adenylate cyclase and protein kinase C activity and by influencing target tissue responsiveness to inflammatory cytokines.

Beyond anti-inflammatory properties, EPA and DHA support endothelial function, enhance insulin sensitivity, and may preserve muscle mass during catabolic stress. However, clinical cautions exist regarding antiplatelet effects and potential bleeding risk. Caution is warranted when administering omega-3 fatty acids to patients receiving anticoagulation therapy, those with pre-existing bleeding disorders, or individuals with documented fish allergies.

In summary, omega-3 fatty acids (EPA and DHA) exert anti-inflammatory effects by displacing arachidonic acid, producing less inflammatory eicosanoids, and generating specialized pro-resolving mediators. In modern practice, omega-3 fatty acids are most commonly administered as the injectable lipid emulsion component of parenteral nutrition (PN) or as a component of a multi-immunonutrient bundle in commercial enteral nutrition formulas.

### 2.4. Other Immunomodulating Nutrients

Several additional nutrients possess immunomodulatory properties. Like glutamine and arginine, nucleotides become “conditionally essential” during rapid cellular proliferation when de novo synthesis pathways cannot meet increased demands for DNA and RNA building blocks. Lymphocytes and macrophages depend heavily on salvage pathways utilizing preformed nucleotides, supporting proliferation, natural killer cell activity, hypersensitivity responses, and gut barrier function [11].

Branched-chain amino acids (BCAAs), including leucine, isoleucine, and valine, possess immunometabolic properties beyond their role as essential amino acids. Leucine activates mTORC1, a central regulator that stimulates muscle protein synthesis while suppressing autophagy. During catabolic stress, BCAAs serve as nitrogen donors for glutamine synthesis and provide alternative oxidative fuel for skeletal muscle, potentially attenuating muscle protein breakdown [45].

Antioxidant micronutrients, including vitamins A (retinol), C (ascorbic acid), and E (tocopherol), along with selenium, maintain redox balance and immune function. Vitamin C functions as a cofactor for enzymes essential for collagen synthesis and wound healing, while vitamin E protects cell membranes from lipid peroxidation. Selenium supports glutathione peroxidase activity, maintains epithelial barrier integrity, and regulates immune cell function. Sulfur-containing amino acids such as cysteine and methionine enhance glutathione concentrations, supporting the body’s primary antioxidant defense system [11].

In summary, nucleotides support immune cell proliferation, BCAAs stimulate protein synthesis and provide alternative fuel during catabolism, and antioxidant micronutrients maintain redox balance and immune function. In modern practice, nucleotides are generally administered as a component of a multi-immunonutrient bundle in an enteral formula. Antioxidant supplements such as vitamin C and selenium are generally administered to treat documented deficiencies but are not routinely given to critically ill patients after several landmark trials demonstrated no benefit and possible harm [41,46].

## 3. Gaps and Challenges in the Clinical Evidence Base

The immunonutrition literature is vast and characterized by numerous methodological limitations, inconsistent and conflicting findings, and persistent knowledge gaps that complicate interpretation, clinical application, and decision-making. There are numerous RCTs and meta-analyses compiling nearly every possible permutation of factors in elective surgery [47,48,49,50,51,52,53,54,55,56,57,58,59,60,61,62], yet definitive conclusions are still lacking due to varying inclusion criteria, treatment regimens, and outcome definitions resulting in substantial heterogeneity across studies, with definitions and assessments of malnutrition varying considerably across biochemical, anthropometric, and composite assessment tools (Table 1). Many trials included mixed populations of malnourished and well-nourished patients without stratification, potentially obscuring true treatment effects. While multiple studies emphasize that severely malnourished patients benefit most from immunonutrition, the precise nutritional thresholds that define this population vary across trials. As a practical reference, the ESPEN 2025 guidelines define high nutritional risk as any of the following: unintentional weight loss exceeding 10–15% within 6 months, BMI below 18.5 kg/m^2^, serum albumin below 30 g/L, or NRS-2002 score ≥ 5 [27]. Patients meeting these criteria represent the subgroup most consistently associated with benefit in the immunonutrition literature, though no trial has prospectively validated these specific thresholds as the optimal cutoffs for patient selection.

Beyond conventional anthropometric and biochemical markers, emerging evidence suggests that inflammatory, oxidative stress, and immune-related biomarkers may help refine patient selection for perioperative immunonutrition. Parameters such as C-reactive protein, neutrophil-to-lymphocyte ratio, prognostic nutritional index, and markers of oxidative stress have been evaluated as potential indicators of nutritional risk and immune competence in hospitalized patients [63,64]. However, none of these biomarkers has been prospectively validated as a selection criterion for immunonutrition in elective surgical populations. Whether inflammatory status, immune function phenotype, or oxidative stress markers can predict individual response to immunonutrition and thereby enable truly personalized nutritional interventions remains an important and unanswered research question.

Beyond patient heterogeneity, immunonutrition products themselves varied substantially across trials, with arginine content ranging from 6.7 to 26 g/L, glutamine content from 0 to 13 g/L, and inconsistent omega-3 fatty acid compositions and nucleotide inclusion. These compositional differences make it impossible to determine whether observed effects result from specific nutrients, particular combinations, or overall nutrient doses. The specific immunonutrient components responsible for clinical benefits and their optimal combinations, doses, and timing remain inadequately characterized. Factorial trial designs testing individual components and combinations could disentangle these effects, but they have not been undertaken. The mechanisms by which immunonutrients exert clinical effects also require better understanding, including whether these nutrients work through complementary mechanisms, justifying their combination. Additionally, some early trials provided 50–60 kcal/kg/day, far exceeding current recommendations and potentially causing metabolic complications rather than benefits [2].

The timing and duration of immunonutrition administration represent another critical source of heterogeneity. Preoperative administration duration ranged from 5 to 7 days, postoperative administration varied from 5 to 10 days, and perioperative protocols combined these approaches differently across studies. For example, one meta-analysis concluded that preoperative immunonutrition alone provided no significant benefit, while both perioperative and postoperative administration reduced complications [52]. However, no adequately powered randomized trial has directly compared all three timing strategies within the same patient population. Additionally, no study has measured whether serum immunonutrient levels remain elevated postoperatively when preoperative-only supplementation ceases at surgery. It remains conceivable that cessation of immunonutrition on the day of surgery results in subtherapeutic or declining levels of circulating pharmaconutrients during the postoperative period when their action may be most valuable, a pharmacokinetic hypothesis that requires testing through measurement of serum concentrations across the perioperative period.

**Figure 3:**
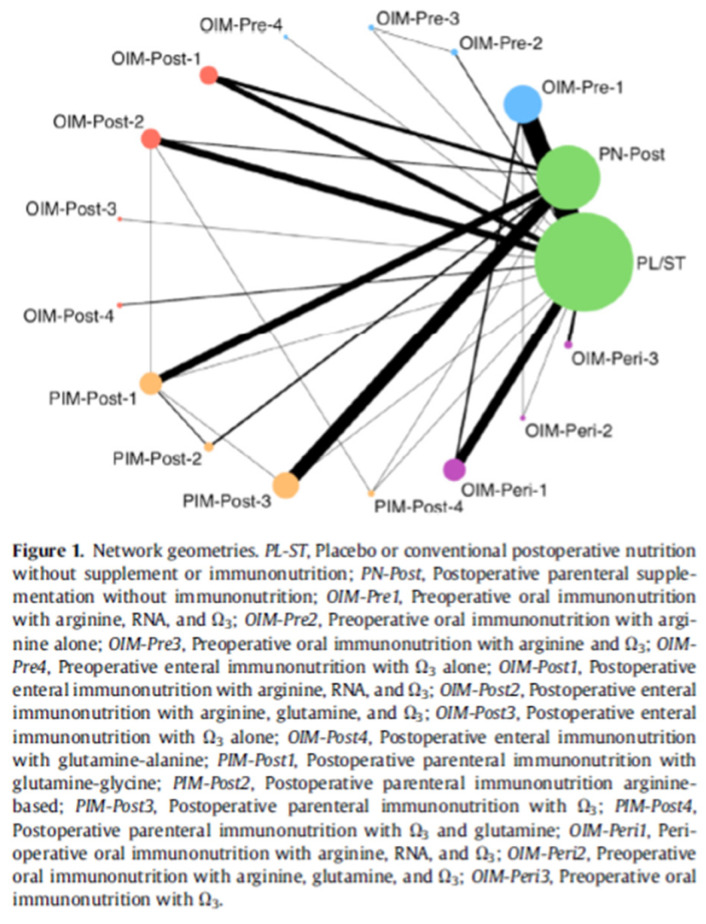
Component network meta-analysis (CNMA) of perioperative immunonutrition interventions. This figure illustrates the relative effects of different immunonutrition components and their combinations on postoperative outcomes, as derived from the CNMA by Ricci et al. [53]. Each node represents a treatment component (arginine, glutamine, omega-3 fatty acids, or nucleotides) or a combination thereof, and the connecting lines indicate direct comparisons between treatments. The size of each node reflects the number of patients included in that treatment arm. CNMA: component network meta-analysis. Adapted from Ricci C. et al. (2023) [53]. © 2023 The Author(s). Published by Elsevier Inc. CC BY 4.0.

Lacking a sufficiently well-designed factorial RCT, the next best level of evidence is a recently completed meta-analysis using sophisticated statistical methods to attempt to account for trial differences [53]. Component network meta-analysis (CNMA) is a technique that can disentangle the impact of each element and suggest the ideal combination of factors (Figure 3). Results of the CNMA are included in the summary recommendations at the end of this section.

Outcome assessment also suffers from a lack of standardization. Slim et al.’s umbrella review found substantial heterogeneity for infectious complications across 20 included meta-analyses, with the majority rated as critically low or low methodological quality by AMSTAR-2 assessment [49]. Probst et al. similarly noted that the beneficial effects of immunonutrition on overall complications vanished after excluding trials at high and unclear risk of bias and that non-industry-funded trials reported no positive effects, while industry-funded trials reported large effects [13]. These findings suggest that inconsistent outcome definitions, variable follow-up durations, and differing control group compositions across immunonutrition trials contribute substantially to the conflicting conclusions in this literature.

The “era” of evidence is also a significant concern, as medical practice has advanced significantly since the 1990s and 2000s. Many immunonutrition RCTs were performed prior to the era of routine lung-protective ventilation, restrictive blood transfusion, hemostatic (“damage control” and “balanced”) resuscitation, judicious fluid administration, and early hypocaloric and moderate feeding. Compared to the event rates of the control arm, immunonutrition intervention arms may no longer experience a significant comparative benefit. Similarly, only a minority of RCTs in elective surgery were conducted within the context of modern minimally invasive surgical techniques and Enhanced Recovery After Surgery (ERAS) protocols [54]. For example, in a recent meta-analysis of immunonutrition in colorectal cancer patients [48], only two [55,56] out of nine studies were conducted in the setting of ERAS.

Industry funding represents another significant concern. It is explicitly noted that many trials demonstrating significant benefits were industry-funded, while non-industry-funded trials with large sample sizes showed fewer clear advantages. When trials with a high risk of bias are excluded in sensitivity analyses, the positive effects of preoperative immunonutrition substantially diminished [52]. This pattern raises concerns about funding bias potentially influencing results and emphasizes the need for independently funded, rigorously designed trials to validate existing findings. Compounding these concerns, multiple studies used non-comparable control groups, with some investigations reporting significant benefits having unusually elevated control group complication rates of 55% to 75% [2]. Some studies compared immunonutrition to no supplementation rather than to isocaloric, isonitrogenous standard nutrition, making it impossible to determine whether benefits resulted from immune-modulating components or simply from adequate calories and protein.

Documentation of actual nutritional intake represents a critical but frequently overlooked methodological requirement. Osland et al. noted that while studies described nutrition goals, few quantified the amounts actually received, forcing assumptions that goals were consistently met. Reduced provision due to feed intolerance, noncompliance with oral supplements, tube-related complications, or protocol deviations may reduce nutrient delivery and confound results [52]. The importance of compliance was demonstrated in a community effectiveness study where the inability to measure patient adherence to the preoperative immunonutrition regimen may have contributed to the lower magnitude of benefit observed compared to controlled trials [57]. In real-world practice settings, factors such as cost, taste preferences, and patient understanding of the intervention’s importance may significantly impact compliance and, consequently, clinical effectiveness [58].

The optimal route for immunonutrient delivery, enteral versus parenteral versus combined, remains inadequately studied despite fundamental differences in physiologic effects. It is well-accepted that enteral nutrition maintains gastrointestinal integrity and has many non-nutritive advantages (including cost) compared to parenteral nutrition. However, feeding intolerance frequently prevents adequate enteral intake in practice.

Economic evaluation of immunonutrition interventions has received insufficient attention despite its importance for healthcare policy and resource allocation decisions. Senkal et al. conducted an early economic evaluation demonstrating cost-effectiveness despite higher product costs [59]. However, their analysis excluded costs associated with prolonged hospitalization and extra intensive care days, likely underestimating the true cost savings. A more comprehensive cost-effectiveness analysis by Chevrou-Séverac et al. addressed methodological limitations by adjusting for disease severity using diagnostic-related group data to isolate costs specifically attributable to complications [60]. Their analysis of 420 gastrointestinal cancer surgery patients demonstrated that complications added an average of CHF 14,949 (€10,901) per patient to hospital costs, while immunonutrition decreased net hospital costs by CHF 1638 to CHF 2488 per patient (€1195 to €1814) across different administration regimens, making it a dominant intervention, both more effective and cost-saving compared to standard care. Sensitivity analyses demonstrated that immunonutrition remained cost-saving even when baseline complication rates were as low as 4–10%. Other investigators have confirmed the overall cost-effectiveness of immunonutrition despite initial upfront costs of the formula [60,61]. Despite these promising findings, economic evaluations from diverse healthcare systems and comprehensive analyses from multiple stakeholder perspectives (hospital, payer, and societal) remain limited.

The generalizability of findings from RCTs to diverse patient populations and real-world clinical settings remains uncertain. Lee et al. noted that their single-institution Korean study included predominantly non-obese, well-nourished patients, questioning whether the results apply to Western populations or different nutritional phenotypes [15]. The gap between efficacy demonstrated in RCTs and effectiveness in real-world practice represents a critical knowledge deficit. A community-based effectiveness study of preoperative immunonutrition reported that the magnitude of benefit observed in this community setting was lower than that reported in randomized controlled trials. The authors attributed this difference to potential compliance issues with the intervention in routine practice, lower baseline complication rates due to concurrent quality improvement initiatives, or both. This discrepancy between tightly controlled trial environments and real-world implementation highlights the importance of effectiveness research to complement efficacy studies [57].

Long-term outcomes beyond the immediate perioperative period have received minimal attention despite their obvious importance to patients and clinicians. None of the reviewed trials examined effects on cancer recurrence, disease-free survival, or overall survival extending beyond 30 days postoperatively. Whether perioperative immunonutrition influences oncologic outcomes, functional recovery, quality of life, or long-term metabolic health remains entirely unknown. Basic science investigations linking biochemical changes to clinical outcomes also remain limited. The disconnection between mechanistic rationale and clinical efficacy requires further investigation through studies correlating immunonutrient serum levels with immune function markers and clinical responses.

Finally, the divergence between guideline recommendations and emerging evidence creates uncertainty about optimal patient care. Current ESPEN guidelines recommend immunonutrition for malnourished surgical patients, yet recent well-designed trials have failed to demonstrate benefit [27]. Lee et al. explicitly concluded that “routine administration of immunonutrition before colon cancer surgery cannot be justified” [15]. This misalignment between guidelines and evidence creates practice variation and raises questions about the strength of the evidence base supporting current recommendations.

To summarize, the current evidence base provides signals of benefit for perioperative immunonutrition in elective surgery, specifically reductions in infectious complications and morbidity, but the certainty of this evidence is limited by the methodological issues described above [49,53,62]. Economic analyses suggest potential cost-effectiveness, though these estimates carry the same methodological caveats. Using GRADE rating criteria and World Health Organization standards for guideline development, current guidelines recommend offering oral/enteral immunonutrition (inclusive of omega-3 fatty acids) to patients with esophageal, gastric, pancreatic, and colorectal cancer undergoing surgical resection, though this recommendation is based on evidence rated as moderate certainty at best [65]. CNMA suggests that oral/enteral multi-component (arginine, glutamine, and omega-3 fatty acid) therapy in the postoperative period is likely the preferred method of delivery based on current evidence, while postoperative PN with omega-3 fatty acids was described by Ricci et al. as yielding favorable results with moderate certainty of evidence. Clinicians should interpret these findings as consistent signals rather than definitive conclusions, given the persistent limitations in study design, heterogeneity, and risk of bias outlined throughout this section [53].

Several important contributions to the evidence base have emerged in 2024 and 2025. Matsui et al. (Ann Surg, 2024) conducted a systematic review and meta-analysis of 48 RCTs across head and neck and gastrointestinal cancer surgery patients, finding high-certainty evidence that immunonutrition reduces total postoperative complications (RR 0.78; 95% CI 0.66–0.93), though the reduction in infectious complications alone did not reach statistical significance in malnutrition-stratified analyses [50]. McKechnie et al. (Colorectal Dis, 2025) performed a systematic review of preoperative immunonutrition specifically in elective colorectal cancer surgery and found a non-significant trend toward reduced anastomotic leak (RR 0.59; 95% CI 0.33–1.04), reinforcing that adequately powered RCTs in this specific context are still lacking [66]. Critically, Pfister et al. (Clin Nutr, 2025) published the first meta-analysis specifically examining immunonutrition in minimally invasive major abdominal surgery (11 RCTs, n = 890) and found no significant benefit for infectious complications, overall complications, or hospital length of stay—a finding that substantially challenges the generalizability of the existing evidence base to the modern minimally invasive surgical setting [51]. Younan et al. (2025) similarly reported that perioperative immunonutrition was not independently associated with anastomotic leak reduction in a well-established ERAS colorectal surgery program, further suggesting that the incremental benefit of immunonutrition may be attenuated when baseline perioperative care is already optimized [67]. Selected landmark trials and recent meta-analyses are summarized in Table 2.

## 4. ESPEN Guidelines 2025

The 2025 ESPEN guidelines on clinical nutrition in surgery provide the overarching framework within which immunonutrition should be applied [27]. These guidelines emphasize that systematic nutritional risk screening, preoperative optimization, avoidance of prolonged fasting, early postoperative oral feeding, and the achievement of adequate protein and calorie targets form the non-negotiable foundation of perioperative care. Immunonutrition represents a selective, adjunctive layer within this broader framework—not a substitute for fundamental nutritional support.

### 4.1. Preoperative Assessment and Optimization

ESPEN recommends universal nutritional screening for all major surgical patients using validated tools such as the NRS-2002 or GLIM criteria [72]. Patients are considered at high nutritional risk if they exhibit any of the following: weight loss > 10–15% over 6 months, BMI < 18.5 kg/m^2^, serum albumin < 30 g/L, or NRS-2002 ≥ 5 [27]. For severely malnourished patients, ESPEN provides a Grade A recommendation to initiate 10–14 days of nutritional optimization prior to elective surgery, even if this requires delaying the procedure [73]. The preferred route is oral supplementation or enteral feeding; parenteral nutrition is reserved for situations where enteral access is not feasible. Modified fasting protocols permitting clear liquids up to 2 h and solids up to 6 h preoperatively, along with preoperative carbohydrate loading, are recommended to reduce insulin resistance and improve metabolic outcomes [27,74].

### 4.2. Postoperative Nutritional Management

ESPEN provides a Grade A recommendation to resume oral feeding within 24 h of surgery in stable patients, prioritizing the enteral route to preserve gastrointestinal integrity and reduce infectious complications [9]. When oral intake remains below 50% of requirements for more than 7 days, enteral tube feeding should be initiated; prophylactic jejunostomy placement should be considered in patients undergoing high-risk upper gastrointestinal procedures [27]. Supplemental parenteral nutrition is indicated within 3–4 days when enteral intake remains below 30% of requirements, with fish oil–containing lipid emulsions preferred when available, given their association with reduced infectious complications and a shorter hospital stay [75].

### 4.3. Immunonutrition Within the ESPEN 2025 Framework

Within this framework, ESPEN recommends offering immunonutrition formulas containing arginine, omega-3 fatty acids, and nucleotides for 5–7 days preoperatively to malnourished patients undergoing major cancer surgery, representing a Grade B recommendation based on consistent meta-analytic evidence [27]. ESPEN explicitly contraindicates glutamine supplementation in critically ill patients with multiorgan failure, based on the REDOXS trial demonstrating increased mortality in this setting—a contraindication that does not apply to elective surgical patients receiving standard-dose multicomponent formulas [24]. The guidelines emphasize that immunonutrition should only be considered after fundamental nutritional care is optimized. When comprehensive ESPEN-aligned perioperative nutrition protocols are implemented within ERAS pathways, 30–50% reductions in postoperative infection rates, reductions in hospital length of stay of 1–2 days, and improved preservation of lean body mass have been reported [76].

## 5. Recommendations for Future Research

Despite decades of research and a plethora of RCTs and meta-analyses, numerous critical questions remain inadequately addressed, requiring focused investigation to optimize perioperative nutritional care. Future research must address methodological limitations through rigorously designed trials while exploring fundamental questions about patient selection, formulation, timing, dosing, and delivery strategies. The path forward demands trials that incorporate clearly defined malnourished populations using validated assessment tools rather than the heterogeneous populations that have characterized much of the existing literature. Prospective validation of nutritional assessment tools to identify patients most likely to benefit represents an urgent priority, as does the investigation of biomarkers beyond traditional anthropometric and biochemical measures. Studies examining whether inflammatory status, immune function, or metabolic phenotypes predict response to immunonutrition could enable truly personalized nutritional interventions tailored to individual patient biology rather than broad population averages.

Control groups in future trials must be truly comparable to intervention groups, utilizing isocaloric and isonitrogenous standard nutrition formulations that differ only in their immunomodulating components. This design element, absent from many existing trials, is essential to determine whether observed benefits result from immune-modulating nutrients specifically or simply from adequate provision of calories and protein. Factorial designs testing individual components, arginine, glutamine, omega-3 fatty acids, and nucleotides, both alone and in combination, would disentangle which specific nutrients drive clinical benefits and whether synergistic effects justify their combination. Dose-response studies for each component could establish optimal dosing thresholds, moving beyond the arbitrary doses used in many commercial formulations to evidence-based targets. Such mechanistic investigations should examine whether these nutrients work through complementary pathways and whether specific surgical populations benefit from particular nutrient profiles rather than generic immunonutrition formulations. An alternative to a complex, multidimensional factorial trial is a “platform trial”, which is a newer adaptive trial design that allows efficient evaluation of multiple simultaneous treatments [77,78]. The RECOVERY (Randomized Evaluation of COVID-19 Therapy) trial, an international collaborative trial enrolling over 50,000 subjects, is an excellent example of the power of a platform trial to evaluate multiple potential treatments such as aspirin [79], azithromycin [80], baricitinib [81], colchicine [82], convalescent plasma [83], dexamethasone [84], hydroxychloroquine [85], lopinavir–ritonavir [86], and tocilizumab [87], among multiple other treatments.

The timing of immunonutrition administration demands direct head-to-head comparison of preoperative-only, postoperative-only, and perioperative strategies within the same patient population using identical formulations and standardized outcome assessments. Current evidence suggests preoperative administration alone provides minimal benefit, while perioperative and postoperative approaches show promise, but no trial has rigorously compared all three strategies. Pharmacokinetic studies measuring serum immunonutrient concentrations across the perioperative period could test the hypothesis that nutrient levels decline when supplementation ceases at surgery, potentially explaining the superiority of continued postoperative administration. Specifically, investigations should measure serum arginine, omega-3 fatty acids, and other pharmaconutrient levels in patients receiving preoperative-only supplementation to determine if therapeutic concentrations persist into the postoperative period when immune modulation may be most critical. Studies should also investigate the minimum effective preoperative duration and optimal postoperative continuation period, questions that remain unanswered despite their obvious clinical relevance.

The route of delivery represents another understudied variable with potentially major physiological implications. Trials comparing enteral, parenteral, and combined routes using equivalent immunonutrient doses could determine whether delivery method affects mechanism of action, tissue uptake, or clinical efficacy. Strategies to optimize enteral tolerance deserve investigation, given that feeding intolerance frequently limits enteral intake in clinical practice despite its theoretical advantages for maintaining gastrointestinal integrity. Evaluation of combined enteral–parenteral approaches requires comparison groups receiving each route independently rather than the uncontrolled designs that have characterized much existing research [5].

Standardized outcome definitions with adequate follow-up duration must become mandatory rather than optional. Common data elements and core outcome sets should be utilized to facilitate future meta-analyses [88,89]. Future trials should explicitly define all outcome variables a priori, assess complications using identical definitions and time windows, and follow patients for at least 30 days postoperatively to capture the full spectrum of treatment effects. Studies examining cancer recurrence, disease-free survival, and overall survival extending beyond 30 days would address the glaring gap in long-term outcome data. Functional outcomes, quality of life, and patient-reported outcomes deserve equal attention to traditional surgical complications, as these patient-centered endpoints may better capture the true value of interventions. Whether perioperative immunonutrition affects long-term metabolic health, body composition, or functional independence remains completely unexplored.

Rigorous blinding protocols and detailed documentation of actual nutritional intake versus prescribed intake throughout the study period should be considered non-negotiable methodological requirements. The field can no longer assume that prescribed nutrition was actually received; quantification of actual delivery must be reported and analyzed. Factors affecting compliance, palatability, cost, convenience, and patient education deserve systematic investigation to understand and improve real-world effectiveness. Studies examining implementation strategies and barriers to guideline adherence could identify actionable targets for quality improvement. Investigation of practice variation and its impact on outcomes would inform whether strict protocol adherence is necessary or whether flexibility in application yields equivalent results. Development of decision support tools for personalized recommendations could help clinicians navigate the complex landscape of patient selection, timing, and formulation choices.

Multicenter international trials examining generalizability across populations with different nutritional phenotypes, body compositions, and baseline inflammatory states would address concerns about the applicability of existing evidence to diverse patient populations. Studies in surgical procedures beyond gastrointestinal operations, extending to orthopedic, cardiac, thoracic, and other major surgical specialties, could determine whether immunonutrition benefits are specific to particular surgical contexts or represent a generalizable perioperative intervention. Community-based effectiveness studies to complement efficacy trials represent a critical need, as Thornblade et al. demonstrated that while the direction of benefit remains consistent when immunonutrition moves from controlled trials to routine practice, the magnitude of effect may be attenuated due to compliance issues, concurrent quality improvement initiatives, and practice variation [57]. Pragmatic trials that account for these real-world factors would provide more realistic estimates of effectiveness for healthcare decision-making, and registry-based trials may facilitate enrollment by embedding the research within usual clinical care [90,91]. Comprehensive cost-effectiveness analyses from multiple perspectives—hospital, payer, and societal—should be integrated into trial design from the outset rather than conducted post hoc. Economic evaluations across different healthcare systems and geographic regions would inform resource allocation decisions and policy development. Budget impact analyses for system-wide implementation would help healthcare organizations understand the financial implications of adopting immunonutrition protocols. Evaluation of patient compliance with oral immunonutrition regimens and strategies to optimize adherence merits particular attention, as compliance may significantly influence real-world effectiveness more than product efficacy. Assessment of how concurrent quality improvement initiatives interact with immunonutrition interventions could explain discrepancies between controlled trials and community practice.

Mechanistic studies correlating immunonutrient serum levels with immune function markers would establish dose–response relationships and identify optimal therapeutic ranges for individual nutrients. Investigation of genomic and proteomic responses to immunonutrition could reveal molecular mechanisms underlying clinical effects and identify biomarkers predicting treatment response. Examination of whether clinical responders demonstrate specific immune or metabolic profiles would enable precision nutrition approaches targeting interventions to patients most likely to benefit. Studies determining optimal biomarkers for monitoring treatment response could enable real-time adjustment of nutritional therapy based on individual patient metabolism rather than fixed protocols.

Finally, the field requires independently funded, well-designed trials to validate or refute findings from industry-sponsored research. The pattern of more favorable results in industry-funded trials compared to investigator-initiated studies raises legitimate concerns about bias that can only be addressed through replication by independent investigators. Only through methodologically rigorous research addressing these fundamental questions, conducted with appropriate funding, sample sizes, and scientific rigor, can the promise of perioperative immunonutrition be fully realized or definitively refuted.

## 6. Conclusions

The evidence regarding perioperative immunonutrition in elective surgery presents a complex landscape. While numerous randomized controlled trials and meta-analyses have been conducted over the past three decades, significant methodological limitations, inconsistent findings, and persistent knowledge gaps complicate clinical decision-making. The evidence base reveals promising signals of benefit in selected populations combined with substantial limitations. Available evidence suggests that immunonutrition may be most beneficial in severely malnourished patients undergoing major gastrointestinal surgery, particularly when administered postoperatively as a multi-component enteral formula combined with an injectable lipid emulsion containing omega-3 fatty acids, though the certainty of this evidence remains moderate at best given the methodological limitations discussed throughout this review. From an economic perspective, immunonutrition may represent a dominant intervention in appropriate patient populations, with analyses suggesting clinical benefit alongside reduced hospital costs, although these estimates derive primarily from older cost-effectiveness studies with methodological limitations. However, focus on specialized immunonutrition should not distract from fundamental perioperative nutritional care, including systematic risk screening, optimization when feasible, early postoperative feeding, and achievement of adequate protein and calorie targets. When implementing immunonutrition in routine practice, clinicians should recognize that real-world effectiveness may be lower than the efficacy demonstrated in randomized trials, with compliance and concurrent quality improvement initiatives potentially influencing outcomes. Future investigators should carefully plan their studies using common data elements and core outcome sets and consider utilizing newer trial designs, such as platform and registry-based trials, to improve generalizability and study efficiency. Only through methodologically rigorous research addressing these fundamental questions can the promise of perioperative immunonutrition be fully realized.

## 7. Clinical Applicability and Practical Guidance

Despite the limitations in the existing evidence base, clinicians must make practical decisions about perioperative immunonutrition in daily surgical care. The following guidance translates available evidence into actionable recommendations, acknowledging inherent uncertainty where it exists.

### 7.1. Patient Selection

The best available evidence supports immunonutrition in malnourished patients—defined by NRS-2002 ≥ 3 or GLIM criteria—undergoing major elective gastrointestinal or oncologic surgery [27,49]. This includes esophagectomy, gastrectomy, pancreaticoduodenectomy, hepatectomy, and major colorectal resection for malignancy. Patients with >10% weight loss in 6 months, serum albumin < 30 g/L, or BMI < 18.5 kg/m^2^ represent the highest-priority group. Routine immunonutrition in well-nourished patients undergoing low-risk or minimally invasive procedures is not currently supported by the evidence: a 2025 meta-analysis of 11 RCTs (n = 890) found that the benefits of immunonutrition observed in open surgery do not translate to the minimally invasive setting [51].

### 7.2. Timing and Duration

Perioperative administration—combining 5–7 days of preoperative oral supplementation with postoperative continuation—is the strategy most consistently supported by meta-analytic evidence [52,53]. Preoperative-only administration has shown inconsistent benefits across studies, while postoperative administration within ERAS pathways has demonstrated reductions in total postoperative complications (RR 0.78; 95% CI 0.66–0.93) in high-quality recent analyses [50]. The minimum effective preoperative duration across most positive trials is 5 days; shorter courses have not been adequately evaluated.

### 7.3. Formula Selection and Route of Delivery

Commercially available multicomponent oral immunonutrition formulas containing arginine (12–15 g/day), omega-3 fatty acids (EPA + DHA ≥ 1.5 g/day), and nucleotides are the most studied and should be the preferred approach for preoperative supplementation [27,53]. The component network meta-analysis by Ricci et al. found that combinations including arginine with omega-3 fatty acids provided the greatest benefit for postoperative infectious outcomes [53]. Enteral administration is strongly preferred over parenteral; intravenous fish oil-enriched lipid emulsions may be considered in patients unable to tolerate enteral immunonutrition [27]. Glutamine-enriched formulas should be avoided in patients with active sepsis, shock, or multiorgan failure per ESPEN 2025 contraindications [24,27].

### 7.4. Practical Implementation Checklist

Before initiating immunonutrition, clinicians should confirm the following: (1) nutritional risk has been formally assessed using NRS-2002 or GLIM; (2) the patient meets threshold criteria for high nutritional risk or confirmed malnutrition; (3) the surgery is major, elective, and involves the gastrointestinal tract or oncologic resection; (4) the surgical approach is not minimally invasive, given the current evidence suggesting attenuated benefit in this setting; (5) patient adherence to preoperative supplementation is realistic and has been counseled; and (6) basic perioperative nutrition elements are addressed within an ERAS framework [27,76]. Immunonutrition should complement, not substitute for, these foundational measures.

## Figures and Tables

**Table 1:** Sources of variability in the immunonutrition literature.

Patient Cohort	Critically Ill Medical ARDS Surgical Trauma Burns Elective Benign Abdominal Cardiac Orthopedic Malignant Colorectal Hepatobiliary ENT Gynecologic Lung Upper GI Open vs. laparoscopic ERAS vs. no ERAS
Nutritional Status	Malnourished Well-nourished Both
Timing	Preoperative Postoperative Both
Administration Route	Included within nutrition Given separately Parenteral Enteral
Ingredients	Glutamine Arginine Omega-3 Selenium Zinc Vitamin C Beta-carotene RNA nucleotides
Dose of Immunonutrient	
Duration of Immunonutrient Treatment	
Year of Publication	Older era Modern era
Baseline Deficiency of Immunonutrient	Yes No
Compliance with Therapy	Yes No Not reported
Source of Funding	Industry Non-industry
Interval to Outcome Assessment	
Primary Endpoint	Mortality Infection Pneumonia Hospital length of stay Anastomotic leak Total postoperative complications Other surrogate endpoints

Abbreviations: ARDS, acute respiratory distress syndrome; ENT, ear, nose, and throat (head and neck surgery); GI, gastrointestinal; ERAS, Enhanced Recovery After Surgery protocol.

**Table 2:** Summary of selected key randomized controlled trials and meta-analyses of perioperative immunonutrition in elective surgery.

Author, Year	Surgery Type	N	Nutritional Status	Intervention	Timing	Key Outcome(s)
Daly et al., 1992 [68]	Upper GI malignancy	85	Malnourished/cancer	Arg/RNA/ω-3 formula vs. standard EN	Postop (7 d)	Reduced infectious complications, shorter LOS; foundational early trial
Senkal et al., 1999 [59]	Elective upper GI surgery	154	Mixed	Multicomponent EN (Arg/ω-3/RNA) vs. standard EN	Postop (7 d)	Reduced late infectious complications (11% vs. 28%, *p* = 0.02); lower costs
Braga et al., 2002 [69]	Major GI cancer (pancreatic, colorectal, gastroesophageal)	150	Malnourished	Arg/Gln/ω-3 formula vs. standard EN	Preop (5 d) + Postop (5 d)	Reduced postop infectious complications; shorter LOS
Gianotti et al., 2002 [70]	Elective GI cancer surgery	305	Mixed	Oral immunonutrition (IMPACT) vs. standard EN	Preop (5–7 d) + Postop	Reduced infectious complications and hospital LOS, greatest benefit in malnourished patients
Turnock et al., 2013 [71]	Head & neck cancer	38	Well-nourished	Oral ω-3/Arg immunonutrition vs. standard	Periop (5 d pre + 5 d post)	No significant benefit; raised questions about patient selection in well-nourished populations
Moya et al., 2016 (SONVI) [56]	Colorectal resection (ERAS)	264	Mixed	Immunonutrition vs. hypercaloric/hypernitrogenous supplement	Preop (7 d) + Postop (5 d)	Reduced total complications (24% vs. 38%); primarily driven by reduction in infectious complications
Hogan et al., 2020 [58]	Pelvic exenteration	81	Mixed	Preop immunonutrition vs. standard polymeric supplement	Preop (5 d)	No significant difference in complications; compliance identified as key confounder
Ricci et al., 2023 (CNMA) [53]	Major abdominal surgery (83 RCTs; n = 7116)	7116	Mixed	CNMA of Arg, Gln, ω-3, nucleotides—all combinations	Periop	Arg + ω-3 combination most effective; glutamine alone not beneficial; industry funding identified as an effect modifier
Matsui et al., 2024 (SR/MA) [50,62]	HAN and GI cancer (48 RCTs)	>4000	Mixed (with/without malnutrition)	Any immunonutrition (Arg/ω-3/Gln) vs. standard nutrition	Periop	Reduced total postop complications (RR 0.78; 95% CI 0.66–0.93); high COE; infectious complications alone not significant
Pfister et al., 2025 (SR/MA) [51]	Minimally invasive major abdominal surgery (11 RCTs)	890	Mixed	Immunonutrition vs. standard nutrition	Periop	No significant benefit for infectious complications, overall complications, or LOS in MIS setting; very uncertain evidence
McKechnie et al., 2025 (SR/MA) [66]	Elective colorectal cancer surgery	Mixed	Mixed	Preoperative enteral immunonutrition vs. conventional nutrition	Preop	Anastomotic leak RR 0.59 (95% CI 0.33–1.04); non-significant trend toward benefit; adequately powered RCTs still lacking

Abbreviations: Arg, arginine; CNMA, component network meta-analysis; COE, certainty of evidence; EN, enteral nutrition; ERAS, Enhanced Recovery After Surgery; GI, gastrointestinal; Gln, glutamine; HAN, head and neck; LOS, length of hospital stay; MIS, minimally invasive surgery; RNA, ribonucleic acid; SR/MA, systematic review and meta-analysis; ω-3, omega-3 polyunsaturated fatty acids.

## Data Availability

No new data were created or analyzed in this study.
